# Clinical Features and Factors Associated with Severity and Fatality among Patients with Severe Fever with Thrombocytopenia Syndrome Bunyavirus Infection in Northeast China

**DOI:** 10.1371/journal.pone.0080802

**Published:** 2013-11-13

**Authors:** Baocheng Deng, Bo Zhou, Shujun Zhang, Ying Zhu, Leqiang Han, Yingzhi Geng, Zhenan Jin, Hongbo Liu, Donglei Wang, Yitong Zhao, Ying Wen, Wei Cui, Ying Zhou, Qiuhong Gu, Cuiming Sun, Xu Lu, Wen Wang, Yu Wang, Chengbo Li, Yanli Wang, Wenqing Yao, Pei Liu

**Affiliations:** 1 Department of Infectious Diseases, the First Affiliated Hospital, China Medical University, Shenyang, Liaoning Province, China; 2 Department of Clinical Epidemiology, the First Affiliated Hospital, China Medical University, Shenyang, Liaoning Province, China; 3 Department of Infectious Diseases, Kuandian Country Hospital, Dandong, Liaoning Province, China; 4 Department of Infectious Diseases, the First Affiliated Hospital, Dalian Medical University, Dalian, Liaoning Province, China; 5 Dalian Municipal Infectious Disease Hospital, Dalian, Liaoning Province, China; 6 Liaoning Province CDC, Shenyang, Liaoning Province, China; 7 Department of Infectious Diseases, Donggang infectious diseases hospital, Dandong, Liaoning Province, China; 8 Department of Infectious Diseases, Liaoning Provincial People's Hospital, Shenyang, Liaoning Province, China; Tulane School of Public Health and Tropical Medicine, United States of America

## Abstract

**Background:**

In 2009, severe fever with thrombocytopenia syndrome virus (SFTSV) was identified as a novel member of the genus phlebovirus in the Bunyaviridae family in China. The detailed clinical features of cases with SFTSV infection have not been well described, and the risk factors for severity among patients and fatality among severe patients remain to be determined.

**Methodology/Principal Findings:**

Clinical and laboratory features of 115 hospitalized patients with SFTSV infection during the period from June 2010 to December 2011 in Northeast China were retrospectively reviewed. We assessed the risk factors associated with severity in confirmed cases and fatality in severe cases by multivariate analysis. One hundred and three (89.6%) of 115 patients presented with multiple organ dysfunction, and 22 (19.1%) of 115 proceeded to the stage of life threatening multiple organ failure. Of the 115 patients, 14 fatalities (12.2%) were reported. Multivariate analysis demonstrated that the independent predictors of risk for severity were: albumin ≤30 g/l (OR, 8.09; 95% CI, 2.58-25.32), APTT ≥ 66 seconds (OR, 14.28; 95% CI, 3.28-62.24), sodium ≤130 mmol/l (OR, 5.44; 95% CI, 1.38-21.40), and presence of neurological manifestations (OR, 7.70; 95% CI, 1.91-31.12). Among patients with severe disease, presence of acute lung injury/acute respiratory distress syndrome (HR, 4.59; 95% CI, 1.48–14.19) and disseminated intravascular coagulation (HR, 4.24; 95% CI, 1.38–13.03) were independently associated with fatality.

**Conclusions/Significance:**

SFTSV infection may present with more severe symptoms and laboratory abnormalities than hitherto reported. Due to infection with a novel bunyavirus, the patients may sufferer multiple organ dysfunction and die of multiple organ failure. In the clinical assessment of any case of SFTS, independent factors relating to prognosis need to be taken into account by clinicians.

## Introduction

In 2009, an emerging infectious disease characterized by severe fever, thrombocytopenia, leukocytopenia and multiorgan dysfunction was identified as being caused by a novel member of the genus phlebovirus in the Bunyaviridae family in China [1]. The disease was first identified as the severe fever with thrombocytopenia syndrome (SFTS) in Central and Northeast China in 2008 [2]. SFTS has an average 12% case fatality rate and even 30% in some areas. Reports that have referred to clinical symptoms of SFTSV infection are either case reports or brief reports, and the detailed clinical features of cases with SFTS virus (SFTSV) infection have not been well described. Several studies have described the factors associated with death in SFTSV patients. However, previously published works assessed risk factors for death by univariate analysis, and the risk factors for severity among SFTS patients and fatality among severe SFTS patients remain to be determined.

We encountered patients with SFTSV infection beginning in June 2010. The major clinical syndromes in critical cases were disturbance of consciousness, severe pneumonia, hemorrhagic signs, coagulopathy, renal function impairment and arrhythmia, which presented with more severe symptoms and laboratory abnormalities than hitherto reported [1–3]. Patients with clinical features of SFTS presented with more serious complications than those of other diseases caused by bunyaviruses (sandfly fever, hemorrhagic fever with renal syndrome, Rift Valley fever and Crimean–Congo hemorrhagic fever) [4–7]. Patients with SFTSV infection in Northeast China are mainly distributed in Liaoning Province. We summarize the clinical features, outcomes and the risk factors associated with severity among SFTS patients and fatality among severe SFTS patients in Liaoning, China from June 2010 to December 2011.

## Methods

### Ethics Statement

Patients all gave written consent to participation in our study. Written informed consent from the guardians on the behalf of participating minors involved in this study was obtained. Permission to perform this study was given by the Ethics Committee of China Medical University. All data analyzed were anonymized.

### Case Definition

Since 2010, an enhanced surveillance and emergency public health response has been implemented in Liaoning to investigate further SFTSV infection. Liaoning Province is located in Northeast China. The total catchment population is 43,746,323. The network included the First Affiliated Hospital of China Medical University, Liaoning Province Center for Disease Control and Prevention, the First Affiliated Hospital of Dalian Medical University, Dalian Municipal Infectious Disease Hospital, Liaoning Provincial People's Hospital and local hospitals in Liaoning Province. Sera obtained from suspected patients during the course of illness were tested for SFTSV at Liaoning Center for Disease Control and Prevention.

A suspected case of SFTSV infection was defined as an acutely ill person with acute onset of fever (≥38.0°C) and other symptoms (e.g. gastrointestinal symptoms, bleeding), epidemiological risk factors (being exposed to ticks or SFTS patients or being a farmer) and laboratory data consisting of thrombocytopenia or leukocytopenia [8]. Confirmed cases of SFTSV infection were defined as those who met the criteria for having a suspected case of SFTS and had also a positive result in a quantitative reverse-transcriptase polymerase chain reaction (RT-PCR), a positive result for IgM antibody to SFTSV, seroconversion of SFTSV-speciﬁc IgG antibody or a 4-fold rise in IgG titer in paired sera. Severe SFTS cases were defined as any person who required admission to an intensive care unit and met at least one of the following criteria: acute lung injury (ALI)/acute respiratory distress syndrome (ARDS), heart failure, acute renal failure, encephalitis, shock, septicemia, disseminated intravascular coagulation (DIC), *or death*.

ALI and ARDS are defined using criteria recommended by the American–European Consensus Conference on ARDS. ALI criteria: (1) Timing: Acute onset; (2) Oxygenation: PaO_2_/FiO_2_ ≤ 300 mmHg (regardless of PEEP); (3) Chest radiograph: Bilateral infiltrates seen on frontal chest radiograph; (4) PAWP: ≤ 18 mmHg when measured or no clinical evidence of left atrial hypertension. ARDS criteria (same as acute lung injury except): Oxygenation: PaO_2_/FiO_2_ ≤ 200 mmHg (regardless of PEEP) [9]. Acute renal failure is defined ≥ 50% increase in serum creatinine and receipt of inpatient acute dialysis. Multiple organ dysfunction and multiple organ failure are defined using criteria as reported by Deitch [10]. Septic shock is defined as sepsis induced hypotension persisting despite adequate fluid resuscitation [11]. Suspected encephalitis is defined as people who developed fever with at least 6 hours of altered mental status, or at least 1 hour of unconsciousness, or seizures, or focal neurologic signs. Encephalitis is defined as the presence of decreased consciousness or seizures or altered mental status or focal neurologic signs, plus at least 2 of the following: (a) fever (>38°C); (b) abnormal cerebrospinal fluid (CSF) examination (pleocytosis >5 white blood cells /mL and/or increased protein content >40 mg/dL) (c) abnormal electroencephalography (EEG) findings compatible with encephalitis (e.g. diffuse or focal slow activity, or periodic lateralized epileptiform discharges); and (d) abnormal results of neuroimaging studies, including computed tomography (CT) and magnetic resonance imaging (MRI) [12,13]. DIC was scored in accordance with the International Society on Thrombosis and Haemostasis scoring system [14]. The scoring system included platelet count (>100×10^9^ cells/L, 0; <100×10^9^ cells/L but >50×10^9^ cells/L, 1; and <50×10^9^ cells/L, 2); elevated fibrin-related marker (no increase, 0; moderate increase, 2; and strong increase, 3) (D-dimer was used); prolonged prothrombin time (<3 s, 0; >3 s but <6 s, 1; and >6 s, 2); and fibrinogen level (>1.0 g/L, 0; <1.0 g/L, 1). A total score of ≥5 was considered to be compatible with overt DIC.

### Diagnostic Tests

The sera obtained from the suspected patients were sent to the laboratory of infectious diseases in Liaoning Province Center for Disease Control and Prevention to be tested for SFTSV infection[15,16]. All serum samples were investigated for SFTSV RNA. IgM and IgG antibody to SFTSV were tested in acute phase sera (within 2 weeks after onset of symptoms). IgG antibody to SFTSV was tested in convalescent phase sera (4 weeks after onset of symptoms) only if results of SFTSV RNA and IgM antibody to SFTSV were both negative in acute phase sera.

### Treatment

If there were no contraindications, the patients received intravenous ribavirin treatment when SFTSV infection was suspected. Fresh frozen plasma was given in patients with DIC depending on their homeostatic state. Platelets were transfused if patients with multiple bleeding sources had a platelet count of less than 20,000 per mm^3^. Antibiotics were administered when secondary bacterial infection was considered. Methylprednisolone for 3–7 days was prescribed in ALI/ARDS patients. Patients were given albumin if serum albumin was less than 25 g/l. Intravenous gamma-globulin (IVGG) was prescribed for cases with severe infection and encephalitis. Granulocyte colony stimulating factor was prescribed when decreased absolute neutrophil count was diagnosed.

### Data Collection

Clinical data on SFTS patients were obtained retrospectively from their medical records. Data including demographic data, underlying medical conditions, clinical presentation and course, and laboratory tests were reviewed by a trained team of physicians and entered in duplicate into a computerized database.

### Statistical Analysis

Results are expressed as mean ± SD, median and as percentages. We performed comparisons between data from patients with non-severe and severe disease in the confirmed cases and data from fatal and non-fatal cases in the severe cases. Means for continuous variables were compared using independent-group Student’s *t* tests for which the data were normally distributed; otherwise, the Mann–Whitney test was used. Proportions for categorical variables were compared using the χ^2^ test, although Fisher’s exact test was used when the data were sparse. Multivariate logistic regression with stepwise backward elimination was used to investigate associations between non-severity and severity in the confirmed cases, and Cox regression was used to model outcomes in the severe cases, allowing a maximum of eight variables for entry in the final model [17]. The starting-point was the onset of complaints and the end-point was the follow-up extended to day 60 after the onset of symptoms. For multivariate analysis, only variables with a *p* value <0.05 were entered into a Cox proportional hazards model and selected using a stepwise selection procedure. Hazard ratio (HR) and 95% confidence interval (95% CI) were computed from estimated parameters of the final regression model. For all analyses, probabilities were 2-tailed, significance was set at *p*<0.05. Results were analyzed using SPSS for Windows version 17.0 (Chicago, IL, USA).

## Results

### Demographics

A total of 136 hospitalized patients were tested for SFTSV from June 2010 to December 2011. There were a total of 115 patients diagnosed with SFTSV infection. Eight (7.0%) of 115 confirmed cases were from southern Liaoning Province, and the remaining confirmed cases were all from eastern Liaoning Province.

**Figure 1:**
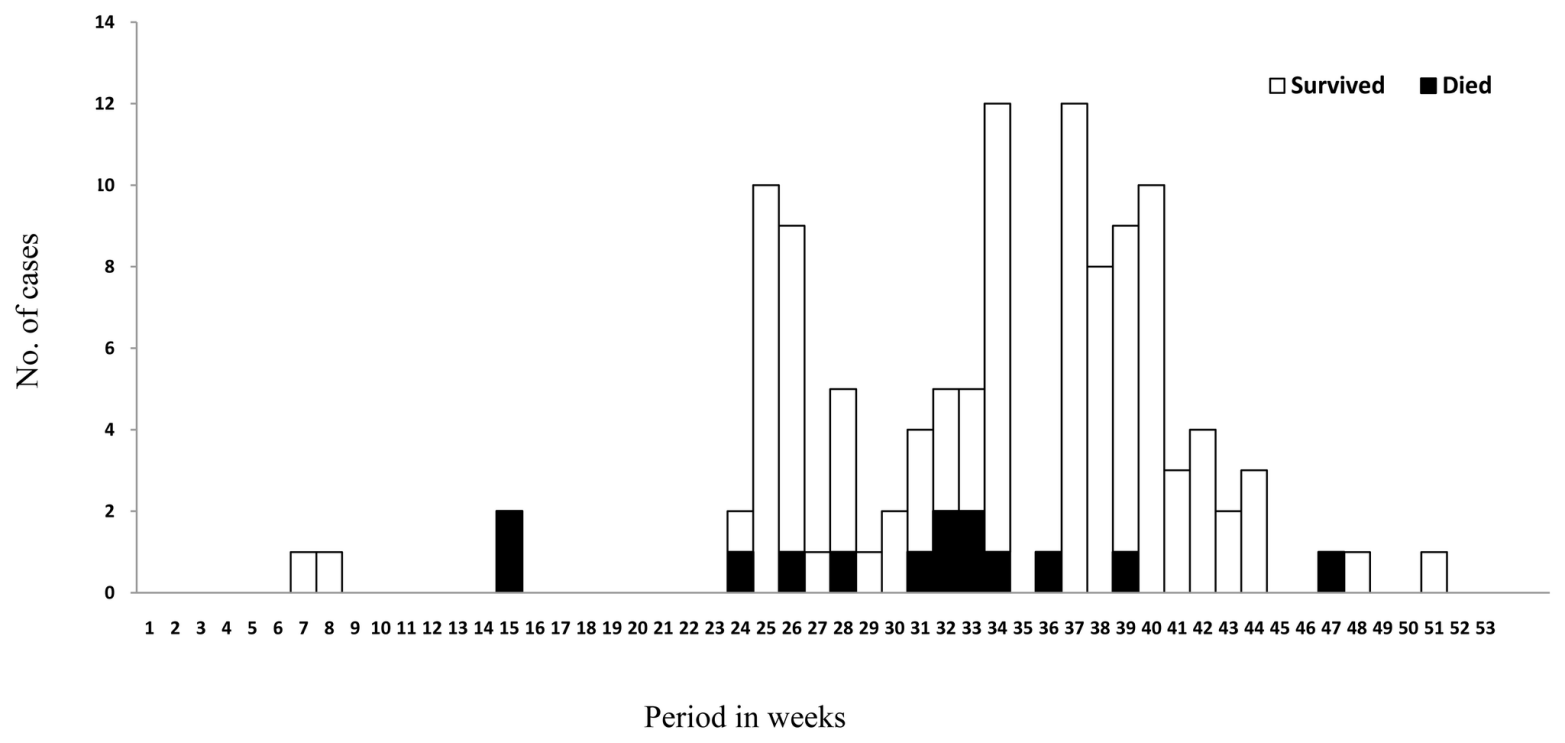
The epidemic curve of cases of SFTSV infection, by weekly periods of symptom onset, starting on June 2010 and continuing through December 2011.

The demographic and epidemiological characteristics, underlying medical conditions and outcomes for the patients with confirmed SFTSV infection are presented in Table 1. Serology and PCR results, characteristics of patients with non-severe and severe disease in the confirmed cases and non-fatal and fatal cases in the severe cases, and complications associated with SFTSV infection are presented in Table 2. Figure 1 shows the epidemic curve of cases of SFTSV infection, by weekly periods, starting June 2010 and continuing through December 2011.

**Table 1:** Characteristics, underlying medical conditions, and outcomes of 115 patients infected with SFTSV in Northeast China (2010–2011).

**Characteristic**	
Male sex - no./total no. (%)	75/115 (65.2)
Age - yr	
Mean	55.0
Range	17 - 89
Age group – no./total no. (%)	
15–30 yr	3/115 (2.6)
31–50 yr	43/115 (37.4)
51–65 yr	51/115 (44.3)
>65 yr	18/115 (15.7)
Occupation – no./total no. (%)	
Farmer	99/115 (86.1)
homeworker	8/115 (7.0)
student	2/115 (1.7)
officer^★^	2/115 (1.7)
worker^▲^	4/115 (3.5)
Confirmed tick bite – no./total no. (%)	7/115 (6.1)
Coexisting conditions – no./total no. (%)	
Hypertension	5/115 (4.3)
Diabetes	7/115 (6.1)
Ischemic heart disease	6/115 (5.2)
Chronic obstructive pulmonary disease	2/115 (1.7)
Asthma	2/115 (1.7)
Chronic hepatitis	5/115 (4.3)
Alcoholic hepatitis	1/115 (0.9)
Chronic hepatitis B	4/115 (3.5)
Cerebrovascular disease	4/115 (3.5)
Seizure disorder	1/115 (0.9)
Renal calculus	1/115 (0.9)
Clinical outcomes	
Incubation period - days^●^	
Median	5
Range	1 - 20
Duration of fever - days	
Median	7
Range	1 - 15
Length of hospital stay - days	
Median	9
Range	1 - 30
Interval between onset and admission - days	
Median	5
Range	1 - 14
Death – no./total no. (%)	14/115 (12.2)
Admission to ICU – no./total no. (%)	41/115 (37.7)
Time from symptom onset to death - days	
Median	11.5
Range	2-30

^★^ Government official working inside.^▲^ Factory worker in town. ^●^ Incubation for transmission through tick bite.

**Table 2:** Characteristics of patients with non-severe and severe SFTS in the confirmed cases and non-fatal and fatal cases in the severe cases admitted with SFTSV infection June 2010-December 2011.

**Characteristic**	**All patients**	**All patients**		***P* value ^a^**	**Severe patients**		***P* value ^b^**
		**Non-severe**	**Severe**		**Non-fatal**	**Fatal**	
	**(n=115)**	**(n=74)**	**(n=41)**		**(n=27)**	**(n=14)**	
Sex, male, no. (%)	75 (65.2)	47 (63.5)	28 (68.3)	0.606	17 (63.0)	11 (78.6)	0.049
Age, year, median (Range)	55.0 (17.0-89.0)	52.5 (18.0-87.0)	59.0 (17.0-89.0)	0.038	61.0 (17.0-80.0)	58.5 (36.0-89.0)	0.762
Duration of fever - days	7.0 (1.0-15.0)	7.0 (2.0-15.0)	8.0 (1.0-15.0)	0.174	8.0 (1.0-15.0)	7.5 (1.0-13.0)	0.763
Length of hospital stay - days, median (Range)	9.0 (1.0-30.0)	9.0 (1.0-16.0)	8.0 (1.0-30.0)	0.148	12.0 (2.0-30.0)	4.5 (1.0-30.0)	0.291
Interval between onset and admission - days	5.0 (1.0-14.0)	5.0 (1.0-10.0)	5.0 (1.0-14.0)	0.428	5.0 (1.0-14.0)	6.0 (1.0-10.0)	0.734
Rout of transmission							
Tick bite, no. (%)	7 (6.1)	2 (2.7)	5 (12.2)	0.095	2 (7.4)	3 (21.4)	0.317
Tick contact (not including tick bite), no. (%)	90 (78.3)	60 (81.1)	30 (73.2)	0.325	22 (81.5)	8 (57.1)	0.140
unknown	18 (15.7)	12 (16.2)	6 (14.6)	0.823	3 (11.1)	3 (21.4)	0.393
Comorbidity, no. (%)	28 (24.3)	13 (17.6)	15 (36.6)	0.023	11 (40.7)	4 (28.6)	0.443
1	23 (20.0)	12 (16.2)	11 (26.8)	0.173	8 (29.6)	3 (21.4)	0.719
2 or 3	5 (4.3)	1 (1.4)	4 (9.8)	0.054	3 (11.1)	1 (7.1)	1.000
Hemorrhagic signs, no. (%)	25 (21.7)	10 (13.5)	15 (36.6)	0.004	6 (22.2)	9 (64.3)	0.008
Neurological manifestations, no. (%)	23 (20.0)	6 (8.1)	17 (41.5)	<0.001	9 (33.3)	8 (57.1)	0.142
Complications, no. (%)							
Pneumonia	33 (28.7)	16 (21.6)	17 (41.5)	0.061	11 (40.7)	6 (42.9)	0.581
Respiratory failure	13 (11.3)	2 (2.7)	11 (26.8)	<0.001	5 (18.5)	6 (42.9)	0.140
ALI/ARDS	7 (6.1)	-	-	-	1 (3.7)	6 (42.9)	0.004
Hepatic insufficiency	95 (82.6)	59 (79.7)	36 (87.8)	0.274	25 (92.6)	11 (78.6)	0.317
Renal insufficiency	32 (27.8)	19 (25.7)	13 (31.7)	0.489	6 (22.2)	7 (50.0)	0.089
Heart failure	5 (4.3)	-	-	-	2 (7.4)	3 (21.4)	0.317
Shock	2 (1.7)	-	-	-	1 (3.7)	1 (7.1)	1.000
Pancreatitis	7 (6.1)	2 (2.7)	5 (12.2)	0.095	4 (14.8)	1 (7.1)	0.645
Encephalitis	4 (3.5)	-	-	-	4 (14.8)	0 (0)	-
Flaccid paralysis	2 (1.7)	0 (0)	2 (4.9)	-	2 (7.4)	0 (0)	-
Aplastic anemia	2 (1.7)	0 (0)	2 (4.9)	-	0 (0)	2 (14.3)	-
Sepsis	8 (7.0)	-	-	-	5 (18.5)	3 (21.4)	1.000
DIC	15 (13.0)	-	-	-	6 (22.2)	9 (64.3)	0.008
Rhabdomyolysis	1 (0.9)	0 (0)	1 (2.4)	-	0 (0)	1 (7.1)	-
Arrhythmia	25 (21.7)	17 (23.0)	8 (19.5)	0.666	4 (14.8)	4 (28.6)	0.411
Use of corticosteroids	7 (6.1)	0 (0)	7 (17.1)	-	1 (3.7)	6 (42.9)	0.004
Use of ribavirin	87 (75.7)	58 (78.4)	29 (70.7)	0.360	20 (74.1)	9 (64.3)	0.719
Use of antibiotics	50 (43.5)	22 (29.7)	28 (68.3)	<0.001	21 (77.8)	7 (50.0)	0.089
Serology and PCR results							
IgM-positive ^c^	86/105 (81.9)	56/64 (87.5)	30/41 (73.2)	0.063	18/27 (66.7)	12/14 (85.7)	0.275
IgG-positive ^c^	32/105 (30.5)	20/64 (31.3)	12/41 (29.3)	0.830	7/27 (25.9)	5/14 (35.7)	0.719
PCR-positive	66/115 (57.4)	38/74 (51.4)	28/41 (68.3)	0.078	20/27 (74.1)	8/14 (57.1)	0.307

^a^ Severe versus non-severe in confirmed cases. ^b^ Fatal versus non-fatal in severe cases. ^c^ Antibody response in acute phase sera.

A total of 98 (85.2%) of 115 patients occurred in summer and autumn. Ticks were commonly found in the patients’ home or work environment. A total of 99 (86.1%) of the 115 patients were farmers living in wooded and hilly areas and working in the fields before the onset of disease. The time between the finding of the tick bite and onset of symptoms in the only 7 patients who had a confirmed history of tick bite ranged from 1 to 20 days. Ninety (78.3%) of 115 patients had a history of tick contact 30 days before onset. The routes of transmission for the remaining 18 (15.7%) of 115 patients were unknown. The median age of the patients was 55.0 years (range, 17–89 years), and 75 (65.2%) of 115 patients were male. In the severe cases, 28 (68.3%) of 41 patients were male, and the median age was 59.0 years, which was significantly higher (*p*=0.038) than that (median, 52.5 years) of non-severe patients. Patients were admitted to different hospitals within 1–14 days (median, 5.0 days) of symptom onset. Twenty-eight (24.3%) of 115 patients of had at least one underling illness, including diabetes, ischemic heart disease, hypertension, chronic obstructive pulmonary disease, asthma, cerebrovascular disease, and chronic hepatitis. The days of hospital stay in patients with underlying illness was significantly longer than that in patients without underlying illness (10.5 vs 8.0, *p*=0.018). The presence of a comorbid condition was associated with severity in the confirmed cases (*p*=0.023), but not with fatality in the severe cases (*p*=0.443). Forty-one (35.7%) patients fulfilled the criteria for severe SFTSV infection. Fourteen (12.2%) patients died, and the median duration from the onset of symptoms until death was 11.5 days (range, 2–30 days). Immune response of SFTSV-specific IgM and IgG in the acute phase sera is not associated with severity or fatality. Among the 105 SFTS patients who had IgM and IgG tested, the median interval between onset and positive result in the initial SFTSV IgM test was 8.0 days (range, 4-14 days). A total of 32 SFTS patients had a positive result for IgG antibody in the acute phase sera, and the median interval between onset and positive result was 12.0 days (range, 8-14 days).

### Course of Illness and Laboratory Indices

Clinical features are presented in Table 3. Fever, fatigue, anorexia, diarrhea, nausea, and myalgia were the most frequent symptoms in all patients. All the patients suffered from fever during the course of the disease (before admission or at admission), most of whom presented with an abrupt onset and severe fever (median temperature, 39.7°C; median duration, 5.0 days). Eighty-nine (77.4%) of 115 patients had fever (≥38.0°C) on the day of admission. The median duration (before and during hospitalization) of fever during the entire disease course was 7.0 days (range, 1–15 days).

**Table 3:** Clinical symptoms of hospitalized patients with laboratory-confirmed SFTS during the course of illness.

**Symptom or sign**	**No./total no. (%)**
Temperature ≥38.0°C (at admission)	89/115 (77.4)
Chill	40/115 (34.8)
Myalgia	47/115 (40.9)
Arthralgia	42/115 (36.5)
Headache	23/115 (20.0)
Fatigue	76/115 (66.1)
Gastrointestinal symptoms	
Anorexia	74/115(64.3)
Nausea	59/115(51.3)
Vomiting	38/115 (33.0)
Diarrhea	65/115(56.5)
Abdominal pain	19/115 (16.5)
Respiratory symptoms	
Cough	27/115 (23.5)
Dyspnea	17/115 (14.8)
Chest pain	6/115 (5.2)
Hemorrhagic manifestations	
Petechiae	13/115 (11.3)
Hematoma on puncture sites	7/115 (6.1)
Hematemesis	2/115 (1.7)
Hematuria	
Macroscopic hematuria	2/110 (1.8)
Microscopic hematuria	20/110 (18.2)
Gingival bleeding	3/115 (2.6)
Conjunctival hemorrhage	3/115 (2.6)
Throat congestion	34/115 (29.6)
Conjunctival congestion	27/115 (23.5)
Hyperemia of face	9/115 (7.8)
Hepatomegaly	7/40 (17.5)
Lymphadenopathy	5/115 (4.3)
Splenomegaly	12/40 (30.0)

The laboratory findings for patients at hospital admission are shown in Table 4 (see Appendix S1 for normal values obtained from laboratory tests in adults). Differences in laboratory characteristics during the course of illness between severe and non-severe cases of SFTSV infection in confirmed cases and between fatal and non-fatal cases of SFTSV infection in severe cases are shown in Table 5 and 6, respectively. Thrombocytopenia (thrombocyte count, <150,000/mm^3^) was the most common abnormality, and 52 (45.2%) of 115 cases were severe (thrombocyte count, <50,000/mm^3^). Leukopenia (leukocyte count, <4,000/mm^3^) was observed in 73 (63.5%) of 115 patients, including 8 (7.0%) of 115 patients with decreased absolute neutrophil count (granulocyte count, <500/mm^3^) and 88 (76.5%) of 115 patients with lymphopenia (lymphocyte count, <1,500/mm^3^). Patients with leukopenia at admission all presented with varying degrees of thrombocytopenia. Thrombocytopenia was seen in 108 (93.9%) patients during the illness course. Additional laboratory tests were performed between days 1 and 5 of hospitalization for 34 hospitalized patients with a normal or elevated leukocyte count, and 21 (61.8%) of 34 patients were shown to have leukopenia. In the present study, a total of 94 (81.7%) of 115 patients showed leukopenia during the illness course. Among 6 patients who underwent bone marrow aspiration, 2 patients were diagnosed with aplastic anemia, 2 patients with decreased proliferation and the remaining 2 patients with reactive hyperplasia. All the 6 patients received intravenous ribavirin with a dose of 500mg every 12 hours for 4-6 days before bone marrow aspiration.

**Table 4:** Laboratory findings of hospitalized patients with laboratory-confirmed SFTS at admission.

**Variable**	**Value**
**Leukocyte count - per mm^3^**	
**Median**	2800
**Range**	600–15,000
**<4000/mm^3^ – no./total no. (%**)	73/115 (63.5)
**>10,000/mm^3^ – no./total no. (%**)	14/115 (12.2)
**Lymphocyte count - per mm^3^**	
**Median**	800
**Range**	100–10,000
**500–1500/mm^3^ – no./total no (%**)	63/115 (54.8)
**<500/mm^3^ – no./total no (%**)	25/115 (21.7)
**Neutrophil count - per mm^3^**	
**Median**	1500
**Range**	100–12,600
**500–1500/mm^3^ – no./total no (%**)	52/115 (45.2)
**<500/mm^3^ – no./total no (%**)	8/115 (7.0)
**Hemoglobin - g/l**	
**Median**	134
**Range**	49-181
**90–120 g/litre – no./total no (%**)	26/115 (22.6)
**60–90 g/litre – no./total no (%**)	8/115 (7.0)
**30–60 g/litre – no./total no (%**)	3/115 (2.6)
**Platelet count - per mm^3^**	
**Median**	55,000
**Range**	10,000–314,000
**50,000–150,000 per mm^3^- no./total no (%**)	53/115 (46.1)
**<50,000/mm^3^ – no./total no (%**)	52/115 (45.2)
**Albumin- g/l**	
**Median**	31.5
**Range**	19 - 49
**Hypoalbuminemia (<35 g/l) – no./total no (%)**	85/115 (73.9)
**Alanine aminotransferase - U/l**	
**Median**	105.0
**Range**	6.0–542.0
**Elevated (>40 U/l) – no./total no (%)**	99/115 (86.1)
**>400 U/l – no./total no (%**)	7/115 (6.1)
**Aspartate aminotransferase - U/l**	
**Median**	157.0
**Range**	13.8 -2,987.2
**Elevated (>35 U/l) – no./total no (%)**	104/115 (90.4)
**>350 U/l – no./total no (%**)	27/115 (23.5)
**Total bilirubin - μmol/l**	
**Median**	11.2
**Range**	2.3 - 171.0
**Elevated total bilirubin (>17.1 μmol/l) – no./total no. (%)**	32/115 (27.8)
**Mildly elevated (<34.2 μmol/l**)** – no./total no.** (**%**)	19/115 (16.5)
**Moderately elevated (34.2–171.0 μmol/l) – no./total no. (%)**	13/115 (11.3)
**Conjugated bilirubin - μmol/l**	
**Median**	3.1
**Range**	0.7 - 140.0
**Elevated conjugated bilirubin (>6.8 μmol/l) – no./total no. (%)**	28/115 (24.3)
**Potassium - mmol/l**	
**Median**	3.7
**Range**	2.2 - 5.9
**<3.5 mmol/l – no./total no (%**)	36/115 (31.3)
**>5.5 mmol/l – no./total no (%**)	1/115 (0.9)
**Sodium - mmol/l**	
**Median**	135.0
**Range**	121.1 - 164.0
**<135 mmol/l – no./total no (%**)	44/115 (38.3)
**>145 mmol/l – no./total no (%**)	1/115 (0.9)
**Chloride - mmol/l**	
**Median**	100.4
**Range**	87.2 - 112.0
**<96 mmol/l – no./total no (%**)	22/115 (19.1)
**>108 mmol/l – no./total no (%**)	5/115 (4.3)
**Calcium^★^**	
**Mean value - mmol/l**	1.95
**Range**	1.03 - 2.99
Hypocalcemia (<2.1 mmol/l) – no./total no. (%)	86/115 (74.8)
Hypercalcemia (>2.6 mmol/l) – no./total no. (%)	1/115 (0.9)
**Creatinine - μmol/l**	
**Median**	75.2
**Range**	4.9 - 370.0
**Elevated creatinine (>104 μmol/l) – no./total no. (%)**	15/115 (13.0)
**Blood urea nitrogen - mmol/l**	
**Median**	4.9
**Range**	1.5 - 35.0
**Elevated blood urea nitrogen (>7.14 mmol/l) – no./total no. (%)**	23/115 (20.0)
**Lactate dehydrogenase - U/l**	
**Median**	457.5
**Range**	20.5 - 11,250.0
**Elevated (>225 U/l) – no./total no (%)**	101/115 (87.8)
**Creatine kinase - U/l**	
**Median**	474.4
**Range**	14 - >23,000
**Elevated (>308 U/l) – no./total no. (%)**	78/115 (67.8)
**>1540 U/l – no./total no. (%**)	15/115 (13.0)
**Elevated creatine kinase MB fraction (>7.2 ng/ml) – no./total no. (%)**	65/97 (67.0)
**Elevated troponin level (>0.4 ng/ml) – no./total no. (%)**	18/31 (58.1)
**Elevated serum myoglobin level (>116 ng/ml) – no./total no. (%)**	1/4 (25.0)
**Elevated serum amylase level (>100 U/l) – no./total no. (%)**	18/48 (37.5)
**Elevated serum lipase level (>60 U/l) – no./total no. (%)**	7/48 (14.6)
**Activated partial-thromboplastin time - s**	
**Median**	42.7
**Range**	18.4 - 137.0
**Prolonged activated partial-thromboplastin time (>33 s) – no./total no. (%)**	93/115 (80.9)
**INR**	
**Median**	1.00
**Range**	0.77 - 4.59
**Elevated INR (>1.15**)** – no./total no.** (**%**)	19/115 (16.5)
**Elevated D-dimer (>0.5 μg/l) – no./total no. (%)**	44/49 (89.8)
**Elevated C-reactive protein (>8 mg/l) – no./total no. (%)**	24/74 (32.4)
**Proteinuria – no./total no. (%)**	64/110(58.2)
**Fecal occult blood – no./total no. (%)**	30/86 (34.9)

★ Corrected serum total calcium.

**Table 5:** Differences in laboratory characteristics between severe and non-severe cases of SFTSV infection in confirmed cases.

**Laboratory characteristics (reference)**	**All cases**	**Non-severe cases (n=74)**	**Severe cases (n=41)**	***P* value**
	**Median (range)**	**Median (range)**	**Median (range)**	
Lowest leukocyte count - per mm^3^	2,600 (600-15,000)	2,800 (680-15,000)	2,400 (600-13,000)	0.112
Lowest neutrophil count - per mm^3^	1,400 (100-12,600)	1,550 (440-12,600)	1,000 (100-9,630)	0.007
Lowest lymphocyte count - per mm^3^	730 (100-10,000)	850 (200-4,000)	600 (100-10,000)	0.073
Lowest platelet count - per mm^3^	50,000 (10,000-312,000)	65,500 (10,000-312,000)	30,000 (14,000-122,000)	<0.001
Highest ALT - U/l	104.0 (6.0-542.0)	91.0 (6.0-488.0)	138.0 (20.4-542.0)	0.024
Highest AST - U/l	179.0 (14.7-2,987.2)	139.9 (14.7-873.0)	311.6 (59.0-2,987.2)	<0.001
Highest AST/ALT ratio	1.9 (0.3-7.8)	1.5 (0.4-5.7)	2.6 (0.3-7.8)	<0.001
Highest LDH - U/l	507.0 (20.5-11,250.0)	394.2 (20.5-6,229.0)	794.8 (136.0-11,250.0)	<0.001
Highest CK - U/l	535.4 (24.7- >23,000.0)	448.8 (24.7-3,847.0)	881.1 (49.0- >23,000.0)	0.002
Longest APTT	45.6 (18.4-180.0)	41.3 (18.4-111.2)	66.6 (27.2-180.0)	<0.001
Highest INR	1.02 (0.79-5.26)	0.97 (0.79-3.06)	1.15 (0.80-5.26)	<0.001
Highest creatinine	82.1 (4.9-370.0)	74.9 (4.9-266.0)	93.0 (46.0-370.0)	0.010
Highest BUN	5.0 (1.5-27.4)	4.8 (1.5-27.4)	6.4 (2.0-25.0)	0.004
Lowest corrected calcium^★^	1.8 (0.7-2.6)	1.9 (0.7-2.6)	1.6 (1.1-2.6)	<0.001
Lowest sodium - mmol/l	135.5 (121.1-164.0)	136.1 (125.0-164.0)	132.9 (121.1-144.6)	<0.001
Lowest albumin - g/l	31.8 (19.0-44.1)	33.1 (22.5-44.1)	28.0 (19.0-38.4)	<0.001
Lowest A/G	1.2 (0.6-2.1)^▲^	1.3 (0.7-2.1)^■^	1.1 (0.6-2.0)	<0.001

^★^ Corrected calcium (mg/dl) = total calcium (mg/dl) + 0.8× [4 − albumin (g/dl)], ^▲^ n=92, ^■^ n=51.

**Table 6:** Differences in laboratory characteristics between fatal and non-fatal cases of SFTSV infection in severe cases.

**Laboratory characteristics**	**Non-fatal cases (n=27)**	**Fatal cases (n=14)**	***P* value**
	**Median (range)**	**Median (range)**	
Lowest leukocyte count - per mm^3^	2,500 (600-11,500)	2,080 (810-13,000)	0.590
Lowest neutrophil count - per mm^3^	1,000 (100-6,400)	1,460 (290-9,630)	0.484
Lowest lymphocyte count - per mm^3^	600 (100-10,000)	640 (240-2,830)	0.755
Lowest platelet count - per mm^3^	28,000 (14,000-122,000)	31,000 (17,000-55,000)	0.286
Highest ALT - U/l	183.9 (32.3-509.0)	121.0 (20.4-542.0)	0.329
Highest AST - U/l	301.0 (59.0-1,197.0)	430.5 (63.0-2,987.2)	0.184
Highest AST/ALT ratio	2.4 (0.3-7.8)	3.5 (2.0-6.2)	0.010
Highest LDH - U/l	606.0 (136.0-6,450.0)	1059.5 (667.2-11,250.0)	0.069
Highest CK - U/l	881.1 (75.0-3,185.0)	721.4 (49.0- >23,000.0)	0.231
Longest APTT - s	53.9 (27.2-104.8)	83.1 (53.0-180.0)	0.006
Highest INR	1.15 (0.8-5.26)	1.20 (0.84-4.59)	0.734
Highest DIC score	4 (2-8)	5 (3-8)	0.035
Highest creatinine - μmol/l	83.5 (54.0-370.0)	113.3 (46.0-264.0)	0.063
Highest BUN - mmol/l	4.9 (2.1-24.9)	8. 7 (2.0-25.0)	0.042
Lowest sodium - mmol/l	132.9 (124.0-140.6)	132.5 (121.1-144.6)	0.391
Lowest corrected calcium^★^- mmol/l	1.6 (1.2-2.0)	1.6 (1.1-2.6)	0.755
Lowest albumin - g/l	28.3 (19.0-37.0)	27.7 (20.0-38.4)	0.587
Lowest A/G	1.1 (0.6-2.0)	1.0 (0.7-1.7)	0.478
Highest CRP - mg/l	5.0 (0.4-150.0)	39.5 (3.3-142.0)	0.028

★ Corrected calcium (mg/dL) = total calcium (mg/dL) + 0.8× [4 − albumin (g/dL)].

**Figure 2:**
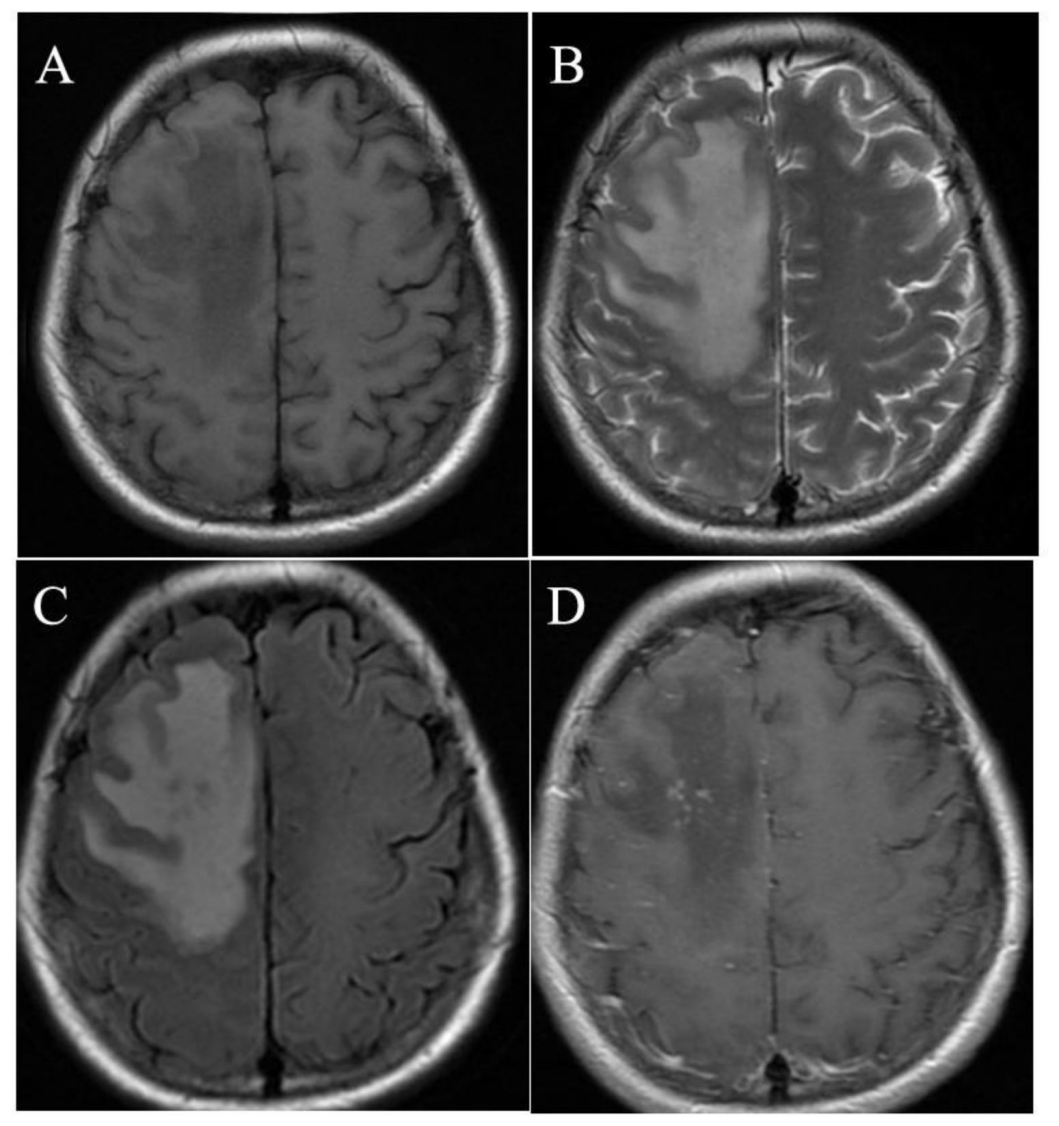
Brain magnetic resonance imaging of a 39-year old patient with encephalitis and acute flaccid paralysis due to SFTSV infection. A. Axial T_1_-weighted image shows hypointense lesion in the right frontoparietal lobes. B. Axial T_2_-weighted image shows the frontoparietal lesion is hyperintense with gyral swelling. C. Axial Flair sequence image shows the lesion is hyperintense with some isointense dots inside. D. Contrast-enhanced T_1_-weighted image shows marked dot-like enhancement in the lesion.

CNS manifestations, CSF laboratory and brain MRI findings are shown in Table 7. Signs of neurological involvement were observed in 23 (20.0%) of 115 cases. Six (5.2%) of 115 patients presented in a state of acute confusion at admission. Generalized tonic-clonic (8 cases) and focal (1 case) convulsions were present in 9 (7.8%) of 115 patients during hospitalization, none of whom had a history of seizures. One patient’s convulsions were attributed to hypocalcemia. Neuroimaging and electroencephalography (EEG) results were normal, and no recurrences were experienced 6 months after onset for the 8 patients with generalized tonic-clonic seizures. Five (62.5%) of the 8 patients with generalized tonic-clonic seizures were in status epilepticus. There was no relationship between convulsions and temperature or blood glucose concentration. Urinary and fecal incontinence were not observed in patients with convulsions. The median Glasgow coma scale at admission was 5 in the 14 coma patients. Tremors involving the upper or lower limb in 13 patients were all static and symmetric. The interval between onset of illness and onset of tremor ranged from 3 to 12 days. Two patients had acute flaccid paralysis (AFP) in both lower limbs and experienced bowel and bladder dysfunction. The paralysis persisted 1 month and 6 months, respectively. Eleven patients had lumbar punctures done. Seven (63.6%) of 11 patients with changes in consciousness had a normal CSF cell count values (≤10 cells/mm^3^), 3 of which died. Neurological manifestations resolved completely in surviving patients during follow-up. Repeat CSF examination was not performed for patients with negative CSF results. Fifteen (13.0%) of 115 patients met the case definition for suspected encephalitis. Four (3.5%) of 115 patients met the case definition for acute encephalitis, all of which recovered without sequelae. Among the 5 patients with comorbidities of cerebrovascular disease and seizure disorder, none had neurological abnormalities during the illness course except that 2 (40%) of 5 patients had transient headache. A 39-year old female classified with AFP also had encephalitis (Figure 2). MRI showed lesions in the right frontoparietal lobes, which showed hypointense lesions on axial T_1_-weighted images and hyperintense lesions with gyral swelling on axial T_2_-weighted images. The CSF cytology results revealed a cell count of 260 cells/mm^3^, polymorphonuclear leukocyte count of 40 cells/mm^3^ and normal protein concentration and glucose level. Electromyography for the patient with AFP and encephalitis showed decreased conduction velocity and undetected sensory function of the tibial and peroneal nerves. The patient recovered fully without any residual neurologic complications, yet follow-up MRI at 24 weeks revealed focal white matter lesions in the right frontoparietal lobes. The other 3 patients with encephalitis who had positive results on MRI all had diffuse lesions in their white matter. Meningeal signs were not observed in this study. A Babinski sign was observed in one patient with encephalitis. There was a significant difference (*p*=0.009) in creatine kinase (CK) level in patients with and without limb tremor. Abnormal changes in EEG, including diffuse and focal slow activity were observed in 3 patients with encephalitis during their acute phase and disappeared in convalescence.

**Table 7:** CNS manifestations, CSF laboratory and brain MRI findings.

**CNS manifestations**	**No./total no. (%)**
Apathy	11/115 (9.6)
Delirium	6/115 (5.2)
Glasgow Coma Scale score 3-8	14/115 (12.2)
Convulsion	9/115 (7.8)
Generalized tonic-clonic	8/115 (7.0)
Focal	1/115 (0.9)
Tremor	13/115 (11.3)
Upper limb tremor	12/115 (10.4)
Lower limb tremor	1/115 (0.9)
Tongue tremor	1/115 (0.9)
Muscle tone	
Normal	100/115 (87.0)
Increased	11/115 (9.6)
Decreased	4/115 (3.5)
Babinski’s sign	1/115 (0.9)
**CSF laboratory findings**	**Value**
Cell count, cells/mm^3^	
≤10 – no./total no. (%)	7/11 (63.6)
10–500 – no./total no. (%)	4/11 (36.4)
Median **-** cells/mm^3^	220
Range **-** cells/mm^3^	150-368
Polymorphonuclear leukocytes in CSF, %	
≤25 – no./total no (%)	3/4 (75.0)
26–50 – no./total no (%)	1/4 (25.0)
Protein concentration, mg/100 ml	
20–40 – no./total no (%)	10/11 (90.9)
41–60 – no./total no (%)	1/11 (9.1)
Glucose level, mg/100 ml	
45-81 – no./total no (%)	11/11 (100)
Bacterial culture	
Negative – no./total no (%)	11/11 (100)
**MRI findings**	**No./total no. (%)**
Lesions	
Negative	8/12 (66.7)
Positive	4/12 (33.3)
White matter	4/4 (100)
T1 hypointense	4/4 (100)
T2 hyperintense	4/4 (100)

Hemorrhagic manifestations including petechia, injection site hematoma, hematuria, hematemesis and fecal occult blood were present in 25 (21.7%) of 115 patients. Twenty-four (96.0%) of 25 patients with hemorrhagic signs had a platelet count <85,000/mm^3^. Only 1 patient with microscopic hematuria had a platelet count of 166,000/mm^3^. All patients with prolonged activated partial-thromboplastin time had thrombocytopenia. With respect to DIC score in the 49 patients, 15 (30.6%) of 49 patients had evidence of DIC, ranging from 5 to 8 (median, 5). No patients with chronic liver disease had DIC. Two patients with encephalitis had a DIC score of 5. Only one patient went into septic shock when DIC occurred.

During the entire course of illness, a total of 93 (80.9%) of 115 patients had complaints of gastrointestinal symptoms, including anorexia, nausea, vomiting, diarrhea or abdominal pain. Elevated serum amylase level was observed in 18 (37.5%) of 48 patients. Seven of 18 (38.9%) patients with three times the upper limit of normal of serum amylase and upper abdominal pain had elevated lipase levels (range, 365.8–2000 U/l ), and were diagnosed with acute pancreatitis due to SFTSV infection. Liver involvement in patients with SFTSV infection was mild to severe, and manifested with raised liver enzymes, such as alanine aminotransferase (ALT) (range, 6.0-542.0 U/l), and conjugated bilirubin (range, 0.7 - 140.0 μmol/l). No liver failure occurred in this study. The liver abnormalities resolved in majority of patients during follow-up for 8 to 12 weeks. Peritoneal effusion on abdominal ultrasound was observed in 1/41 (2.4%) patients who underwent abdominal ultrasonography, accompanied by pericardial effusion.

Decline in creatinine clearance rate (Ccr) was observed at the time of admission in 15 (13.0%) of 115 patients. The interval between symptom onset and hospital admission ranged from 3 hours to 10 days in patients with reduced Ccr at admission. Most patients in the study had mild renal function impairment (51≤ Ccr ≤70 ml/min). Only 5 (4.3%) of 115 patients with renal function impairment had severe renal function impairment (Ccr ≤30 ml/min) at admission (range, 19.6 - 28.3 ml/min). During the whole illness course, renal function impairment (Ccr ≤70 ml/min) was observed in 32 (27.8%) of 115 patients, 10 (31.3%) of whom presented with CK level >200 mmol/L. Serum ​​myoglobin was tested in 5 patients, and only 1 had an elevated serum myoglobin. One patient with a Ccr of 19.6 ml/min had a CK value exceeding the maximum (>23,000 U/L) and ​​myoglobin value (>1000 U/L), and the renal injury was considered to be associated with rhabdomyolysis [18]. A marked increase (>1540 U/l) in the creatinine kinase level occurred in 15 (13.0%) of 115 patients.

Among other, less common signs, chest pain was reported in 6 (5.2%) of 115 patients. Radiographic findings of patients with SFTSV infection are shown in Table 8. Of the 98 patients who underwent chest radiography or chest computed tomography (CT), 33 (33.7%) had abnormalities suggestive of pneumonia. Of the 33 patients with pneumonia, 12 (36.4%) had ground-glass opacities on chest CT or radiography that were distributed diffusely. Other radiographic findings included bilateral infiltrates (n=10), an infiltrate limited to one lobe (n=5), and multilobar infiltrates limited to 1 lung (n=6). Bacterial infections were detected by blood culture in 6 patients with pneumonia, including 2 patients with ALI/ARDS (*E. coli* and *Candida krusei*, respectively). Results of sputum culture were all negative. Most patients received antibiotics near the time of culture collection, which could have reduced the diagnostic sensitivity. Respiratory failure (PaO2 ≤60 mmHg) was observed in 13 (44.8%) of 29 patients on whom blood gas analysis was performed, and 5 (38.5%) of 13 had ARDS due to SFTSV or secondary bacterial or fungal infection, and required pressors and mechanical ventilation. The median age of patients with respiratory failure was 62 years (range, 36–78 years). All patients with pneumonia received 1–3 antibiotics during the course of the disease.

**Table 8:** Radiographic findings of patients with SFTSV infection.

**Radiographic findings**	**No./total no. (%)**
Normal	54/98 (55.1)
Abnormalities	44/98 (44.9)
Local patchy shadowing – no./total no. (%)	21/98 (21.4)
Ground-glass opacities – no./total no. (%)	12/98 (12.2)
Pleural effusion	10/98 (10.2)
Unilateral – no./total no. (%)	1/98 (1.0)
Bilateral – no./total no. (%)	9/98 (9.2)
Pericardial effusion on chest CT or echocardiography	5/35 (14.3)

Five (4.3%) of 115 cases had acute left ventricular failure during the illness course. Eighteen (58.1%) of 31 patients who had troponin levels checked had an increased troponin level. Twenty-five (23.6%) of 106 patients who underwent electrocardiography (ECG) showed arrhythmia including sinus bradycardia (n=5), supraventricular arrhythmias (n=8), premature ventricular beats (n=5), ventricular fibrillation (n=1), atrial fibrillation (n=6), and atrioventricular block (n=2). Eight (7.5%) of 106 patients had changes in T wave, which were flat, diphasic, or inverted without ST-segment abnormality. Elevated troponin level ranging from 0.407 to 3.55 ng/ml was observed in 18 (58.1%) of 31 patients who had checked troponin levels, including 2 patients with a comorbidity of ischemic heart disease. Five (14.3%) of 35 patients who had chest CT or echocardiography had mild pericardial effusion, and none of these patients had characteristic ECG changes in acute pericarditis. Clinical evidence of paradoxical pulse and decreased systemic arterial pressure were not observed in patients with pericardial effusion.

Eight (7.0%) of 115 patients had confirmed bacteremia. The pathogens were identified, including *E. coli* (n=2), *Staphylococcus haemolyticus* (n=1), *Klebsiella pneumonia* (n=1), *Listeria monocytogenes* (n=1), *Candida albicans* (n=1), *Candida krusei* (n=1), and *Acinetobacter baumannii* (n=1). Hospital–acquired infections occurred in 4 (50%) of the 8 patients, and the pathogens were *Candida albicans*, *Candida krusei*, *Acinetobacter baumannii and Klebsiella pneumonia*, respectively.

## Treatment

Intravenous ribavirin was used in 87/115 (75.7%) of patients. There was no significant difference between the laboratory results for patients who received ribavirin and for those who did not. In patients with ribavirin prescribed, the median interval between symptom onset and initial use of ribavirin was not significantly different between severe cases and non-severe cases, and among severe patients with ribavirin prescribed there was no significant difference between fatal cases and non-fatal cases. Ninety-two percent of these individuals received ribavirin with a dose of 500mg every 12 hours within the first 24 hours of hospitalization. The median treatment course of ribavirin was 4 days (1-7days). There was no significant difference (*p*>0.05) in clinical outcome, including duration of fever, length of hospital stay and death, between patients who received ribavirin and for those who did not. Toxicity associated with ribavirin was noted. Three of 87 (3.4%) experienced a decrease in hemoglobin level of at least 2 g/dL accompanied by 1.5-fold increase in bilirubin after ribavirin was initiated.

### Outcomes

Most patients with SFTSV infection had a good outcome. One hundred and three (89.6%) of 115 patients presented with multiple organ dysfunction. Twenty-two (19.1%) of 115 proceed to the stage of life threatening multiple organ failure. There were 14 (12.2%) of 115 patients who died of multiple organ failure. Clinical features and laboratory tests at admission associated with fatality performed by univariate analysis are present in Table 9. Those with hemorrhagic signs, coagulopathy, severe thrombocytopenia, severe kidney injury, hypoalbuminemia, higher lactate dehydrogenase level at admission tended to have a poorer outcome.

**Table 9:** Laboratory tests and clinical symptoms in fatal and non-fatal cases on admission.

	**Fatal**	**Non-fatal**	***P* value**
	(N=14)	(N=101)	
**Laboratory tests**	Median (range)	Median (range)	
Platelet count - per mm^3^	33,500 ( 17,000–314,000)	61,000 (10,000–312,000)	0.010
Aspartate aminotransferase - U/l	279.5 (46.0–2987.2)	153.0 (13.8–2185.0)	0.040
Lactate dehydrogenase - U/l	911.5 (86.5–11,250.0)	457.5 (20.5–6450.0)	<0.001
Blood urea nitrogen - mmol/l	7.15 (2.0–25.0)	4.8 (1.5–35.0)	0.004
Creatinine - μmol/l	101.2 (46.0–264.0)	73.8 (4.9–370.0)	0.005
Albumin - g/l	27.0 (20.0–34.0)	31.8 (19.0–49.0)	0.001
INR	1.13 (0.83–4.59)	1.00 (0.77–2.57)	0.042
Activated partial-thromboplastin time - s	70.2 (42.7–137.0)	42.7 (18.4–104.8)	<0.001
**Clinical symptoms**	No. (%)	No. (%)	
Hemorrhagic signs	8 (57.1)	5 (5.0)	<0.001

Tables 5 and 6 show the results of univariate analysis of the association between possible risk factors in the confirmed cases and severe cases, respectively. Levels of ALT, aspartate aminotransferase (AST), AST/ALT ratio, lactate dehydrogenase (LDH), CK, international normalized ratio (INR), creatinine, and blood urea nitrogen (BUN) were significantly higher, and activated partial thromboplastin time (APTT) was significantly longer among patients with severe disease in the confirmed cases, and neutrophil count, platelet count, levels of total calcium, sodium, albumin and albumin globulin ratio (A/G) were significantly lower among patients with severe disease. As shown in Table 10, in the multivariate full logistic regression model factors with independent significant associations with severity in the confirmed cases were: albumin ≤30 g/l (OR, 8.09; 95% CI, 2.58-25.32), APTT ≥ 66 seconds (OR, 14.28; 95% CI, 3.28-62.24), sodium ≤130 mmol/l (OR, 5.44; 95% CI, 1.38-21.40), and presence of neurological manifestations (OR, 7.70; 95% CI, 1.91-31.12).

**Table 10:** Multivariate analyses of risk factors associated with disease severity in confirmed cases due to SFTSV infection June 2010-December 2011.

**Variable**	**OR**	**(95% CI)**	***P* value**
**Lowest albumin ≤30 g/l**	**8.09**	**2.58-25.32**	**<0.001**
**Longest APTT ≥66 s**	**14.28**	**3.28-62.24**	**<0.001**
**Lowest sodium ≤130 mmol/l**	**5.44**	**1.38-21.40**	**0.015**
**Neurological manifestations**	**7.70**	**1.91-31.12**	**0.004**

Among patients with severe disease during the whole disease course, higher AST/ALT ratio and DIC score, prolonged APTT, elevated levels of BUN and C-reactive protein (CRP) were associated with fatality in univariate analysis. Multivariate logistic regression analysis was not attempted because of the small number of severe patients. Cox proportional hazards model was used for fatality analysis in the severe cases. The time starting-point was onset of symptoms and the time end-point was follow-up extended to day 60 after the onset of symptoms. As shown in Table 11, presence of ALI/ARDS (HR, 4.59; 95% CI, 1.48–14.19) and DIC (HR, 4.24; 95% CI, 1.38–13.03) were independently associated with mortality in the severe cases.

**Table 11:** Cox regression analysis of survival in 41 patients with severe SFTSV infection June 2010-December 2011.

**Variable**	**HR**	**95% CI**	***P* value**
ALI/ARDS	4.59	1.48-14.19	0.008
DIC	4.24	1.38-13.03	0.012

## Discussion

Current knowledge of SFTS is limited. We describe herein a cohort of 115 hospitalized patients with SFTSV infection, and examined risk factors for disease severity among SFTS patients and fatality among patients with severe SFTS by multivariate analysis. Similar to patients described before, fever, gastrointestinal symptoms and fatigue were common clinical features of SFTS [1,19]. The major clinical syndromes in severe cases were disturbance of consciousness, arrhythmias, heart failure, ALI/ARDS, and DIC, in addition to fever, thrombocytopenia and leukopenia, which were different from those reported previously [1,20]. The mortality is similar to that in various studies.

Although SFTS can occur in a non-epidemic area [21], SFTS is mostly seen in endemic areas. The most affected population is farmers. Most of these cases were living in hill areas, and ticks were commonly found in most patients’ home and work environments [22]. It has been confirmed that SFTSV can be transmitted to humans by tick bites, contact with blood from SFTS patients, and personal contact [23,24]. In endemic areas, SFTS occurs most frequently in the summer and autumn seasons, which are suitable for tick activity and consistent with the increasing outdoor activities that increase the risk for exposure to ticks [25]. To date, the true incubation period of the virus has not been established definitively. It has been reported that the incubation period for transmission through tick bite in most SFTS patients ranges from 5 days to 2 weeks [23]. In the present study, the median incubation period for transmission through tick bite in the 7 patients who had a definite history of tick bite was 5 days. However, the delay between the tick bite and the onset of symptoms in 3 of these 7 patients ranged from 2 to 3 weeks, which suggests that the incubation for transmission through tick bite of SFTSV infection may be longer. There were no family cluster or person-to-person transmission of infections by SFTSV reported in this study, so we know little about the incubation of SFTSV infection through person-to-person transmission.

The hallmark laboratory findings at admission in the present study were leukopenia and thrombocytopenia. Two deaths with pancytopenia presented with empty bone marrow, which was consistent with a diagnosis of aplastic anemia. Hemophagocytic lymphohistiocytosis associated with SFTSV infection was observed in 2 patients in Zhejiang Province in East China recently[26]. Four (66.7%) of 6 bone marrow smears done on our patients revealed decreased proliferation of hematopoietic progenitor cells, and further studies are required to elucidate the pathogenic mechanism.

The liver plays a central role in disease pathogenesis of SFTSV infection [3]. The elevated markers of liver damage, such as ALT, AST and LDH were observed in majority of the 115 patients, with AST levels typically higher than ALT levels, and were especially pronounced among patients with severe disease. We found that markers of liver damage, such as ALT and AST, were associated with poor prognosis, which is similar to Crimean-Congo hemorrhagic fever (CCHF) and dengue hemorrhagic fever (DHF) [27,28]. In this study, AST levels were disproportionately higher than ALT levels in SFTS patients, which were dramatically higher (2–6.2 times higher) in fatal than in non-fatal cases. Some of patients with elevated markers of liver damage had complications, such as pancreatitis, renal insufficiency, rhabdomyolysis [18], sepsis and shock. This suggests that elevated AST/ALT ratio is not only associated with liver damage, but also with damage to other tissues, such as muscle, kidney, and pancreas, which could be a direct result of virus infection (and injury) and/or an indirect consequence of cell necrosis [29].

Central nervous system (CNS) manifestation was observed in one-fifth of cases at admission. Due to not all patients with disturbance of consciousness having had a CSF count performed and repeat CSF examination was not performed for patients with negative results on CSF examination, encephalitis was probably underestimated. Evidence of encephalitis was not found in 19 (82.6%) of 23 patients with CNS manifestation, so these cases may have been due to toxic-metabolic encephalopathy, but further workup would be necessary to completely rule out encephalitis. Abnormal findings of MRI images in the 4 patients with encephalitis were consistent with acute inflammation, and CSF features were also more consistent with acute viral encephalitis. Marked elevation of CK level was observed in patients with limb tremor. The only patient with acute kidney failure and limb tremor was associated with rhabdomyolysis. The significant difference in CK level in patients with and without limb tremor suggests that muscle injury associated with SFTSV infection may also play a role in limb tremor, as well as central or peripheral neurologic involvement, renal or hepatic involvement.

The frequency of lymphadenopathy, compared with that reported by Yu et al, (4.6% vs 27.2%), was probably underestimated in our study [1]. Prominent flushing of the head, neck, and upper chest was rarely observed in patients with SFTSV infection, which is different from hemorrhagic fever with renal syndrome. Hemorrhagic signs are observed in SFTS patients. Most patients with hemorrhagic signs showed microscopic hematuria, fecal occult blood, occult blood in their vomitus and petechiae. Severe hemorrhagic signs, such as gastrointestinal bleeding, macroscopic hematuria and hematoma, are commonly observed in patients with DIC and aplastic anemia due to SFTSV infection, which was responsible for 3 deaths in the present study.

Factors related to death from SFTSV infection have previously been assessed in only a few studies [2,3,19]. Zhang et al. reported that the fatal outcome was associated with age, high serum AST levels, pronounced coagulation disturbances, and high levels of acute phase proteins among 49 cases of SFTS [3]. Xu et al. have found that elderly patients and those with underlying diseases, neurological manifestations, coagulopathy, or hyponatremia tend to have a poor outcome [20]. In another study, we found that the severity and clinical outcome in patients with novel bunyavirus infection are associated host immune responses [30]. SFTSV also has played a direct role in these fatalities [19,31]. However, these results were assessed by univariate analysis. To the best of our knowledge, no study has reported the risk factors associated with severity in SFTS patients. With a multivariate logistic regression model to control confounding and estimate the direct effects of several possible causal risk factors, we found that hypoalbuminemia, prolonged APTT, hyponatremia, and presence of neurological manifestations were significantly associated with severity in SFTS patients. Among patients with severe SFTS, high serum AST levels, pronounced coagulation disturbances, similar to the previous study, were also associated with fatality, but Cox proportional hazards model showed that the presence of ALI/ARDS and DIC were independent predictors of fatality among patients with severe SFTS.

Besides leukopenia, the hallmark laboratory finding in SFTS patients was thrombocytopenia. Patients with severe disease also had lower platelet counts than those with non-severe disease in the confirmed cases (*p*<0.001). DIC score was ascertained among patients with severe disease, 15 of which were diagnosed with DIC. DIC has previously been shown to be more prominent in patients with fatal CCHF [32]. DIC was also an independent prognostic factor among patients with severe SFTS. The coagulation disturbances may be secondary to endothelial damage, DIC, or decreased production of coagulation factors due to acute hepatic damage, which is similar to other viral hemorrhagic fevers such as CCHF [3].

 Nearly one third of patients had renal function impairment, most of which was mild. Electrolyte abnormalities were commonly observed. Hyponatremia is considered the most prominent change in serum electrolytes in Hantavirus infection, but is rarely serious [33,34]. Acute or symptomatic hyponatremia can lead to significant rates of morbidity and mortality [35,36]. We found that moderate hyponatremia was an independent risk factor for severity in SFTS patients. It has been reported that extremely low (<0.8 mmol/l) concentration of calcium ions is an independent predictor of mortality in critical illness [37]. Hypocalcemia was more serious in fatal than non-fatal cases. In this study, the corrected total serum calcium, not ionized calcium, was investigated. Total serum calcium levels are not reliable, therefore, we can’t assess the association between prognosis and serum calcium. Seven of 11 patients with changes in consciousness had a normal CSF cell count, which suggested other causes, such as electrolyte or other metabolic disturbance may be associated with neurological manifestations.

Currently, there is no specific antiviral therapy for SFTSV infection. Supportive therapy, including intensive monitoring to guide volume is the most essential part of case management. Ribavirin is reported to be effective for treating CCHF infections and hemorrhagic fever with renal syndrome [7,38]. In this study, ribavirin had no influence on clinical outcome in SFTS patients. Due to the retrospective nature of our study, the efficacy of ribavirin in the treatment of SFTS is not clear. Adverse effect associated with ribavirin was also observed. Corticosteroids increase the risk of developing critical disease from viral infections. The use of corticosteroids for the treatment is controversial in SFTS patients with ALI/ARDS. Host immune responses play an important role in determining the severity and clinical outcome in patients with infection by SFTSV [3,30]. Corticosteroids can suppress the cytokine storm. We hoped that the progression of pulmonary disease would be stopped by corticosteroids, which was proved in patients with severe acute respiratory syndrome [39]. There was no control group, so we can’t determine whether SFTS patients with ALI/ARDS can benefit for administration with corticosteroids. A prospective case-control study is required to evaluate the efficacy of ribavirin and corticosteroid treatment.

The present study had several limitations characteristic of a retrospective study. First, symptoms and laboratory data may not have been recorded comprehensively. Second, patients with milder illness who may have not presented to the hospital and children (<14 years old) were not included, so some of these patients were filtered out. The group may not be representative of hospitalized patients who may not have been tested for SFTSV in 2010. Because there are currently no data on how long SFTSV IgM antibody can persist, the case definition couldn’t exclude patients with positive results for SFTSV IgM who may have had other reasons for disease and have been incidentally found to have IgM. Third, the sample size is not large enough to investigate the association between survivors and fatalities among severe patients using multivariate logistic regression analysis. These may have affected the results.

In conclusion, this study in Northeast China reveals that SFTSV infection may present with more severe symptoms and laboratory abnormalities than hitherto reported, which were poorly characterized or incompletely documented before. Due to infection with a novel bunyavirus, the patients may sufferer multiple organ injury and die of multiple organ failure. There are no pathognomonic cluster of signs and symptoms that indicate SFTSV infection, but surely after reviewing the manifestations of these patients, a more helpful conclusion about the possible manifestations of SFTSV infection in hospitalized patients can be achieved. This would be a useful contribution for clinicians who might suspect, test, report SFTSV infection and assess SFTSV infection in future cases. In the clinical assessment of any case of SFTS, findings relating to prognosis, including hypoalbuminemia, prolonged APTT, hyponatremia and presence of neurological manifestations, DIC and ALI/ARDS, need to be taken into account by clinicians. Efficacy of ribavirin and corticosteroids in SFTS patient remained to be determined.

## Supporting Information

Appendix S1 (DOC)
