# Supplementary material for: Clinical Features and Factors Associated with Severity and Fatality among Patients with Severe Fever with Thrombocytopenia Syndrome Bunyavirus Infection in Northeast China
Source: PLoS One. 2013 Nov 13;8(11):e80802. doi: 10.1371/journal.pone.0080802 (PMC3827460; doi:10.1371/journal.pone.0080802)
Supplement: Appendix S1 — (DOC) [file pone.0080802.s001.doc]

**Normal values obtained from laboratory tests in adults:**

Leukocyte count: 4,000–10,000/mm^3^; lymphocyte count: 1,500–4,000/mm^3^; neutrophil count: 1,500–7,000/mm^3^; platelet count: 150,000–350,000/mm^3^; ALT: 5–40 U/l; AST: 8–35 U/l; LDH: 135–225 U/l; CK: 39–308 U/l; creatinine: 59–104 μmol/l; BUN: 2.85–7.14 mmol/l; sodium: 135–145 mmol/l; calcium: 2.1–2.6 mmol/l; albumin: 35–55 g/l; CRP: 0–8 mg/l. APTT: 23–33 s; INR: 0.82–1.15.
